# Newly discovered circRNAs in rheumatoid arthritis, with special emphasis on functional roles in inflammatory immunity

**DOI:** 10.3389/fphar.2022.983744

**Published:** 2022-10-07

**Authors:** Zhenyu Li, Jianpeng Wang, Yudong Lin, Jihong Fang, Kang Xie, Zhiye Guan, Hailong Ma, Liang Yuan

**Affiliations:** ^1^ Wannan Medical College, Wuhu, China; ^2^ The First Affiliated Hospital of Anhui Medical University, Anhui Medical University, Hefei, China; ^3^ Department of Pediatric Orthopedics, Anhui Provincial Children’s Hospital, Hefei, China

**Keywords:** CircRNAs, rheumatoid arthritis, inflammatory, ra, immune

## Abstract

Circular RNA (circRNA) is a unique type of endogenous RNA. It does not have free 3 ′or 5′ ends, but forms covalently closed continuous rings. Rheumatoid arthritis (RA) is a common chronic autoimmune joint disease, characterized by chronic inflammation of the joint synovial membrane, joint destruction, and the formation of pannus. Although the pathogenesis of rheumatoid arthritis remains incompletely understood, a growing amount of research shows that circRNA has a close relationship with RA. Researchers have found that abnormally expressed circRNAs may be associated with the occurrence and development of RA. This article reviews the inflammatory immune, functions, mechanisms, and values of the circRNAs in RA to provide new ideas and novel biomarkers for the diagnosis and treatment of RA.

## 1 Introduction

Rheumatoid arthritis (RA) is a common chronic autoimmune joint disease, characterized by chronic inflammation of the joint synovial membrane, joint destruction, and the formation of pannus. Today, the global prevalence of RA is approximately 1% and it is more common in women, with many studies reporting a 3–5-fold higher prevalence of RA in women than in men (Scott et al., 2010; Aletaha and Smolen, 2018; Ferreira et al., 2019). Suppose patients with RA are not diagnosed and treated appropriately in the early stage. In that case, it will bring significant hidden troubles to patients and may eventually lead to joint deformity and loss of function, seriously affecting patients’ emotions, quality of life and social function, etc., (Hassan et al., 2019; Hitchon et al., 2020). In recent years, the development of biological agents has exerted significant influence on the treatment of RA, but so far, RA still cannot be cured, which may be related to the pathogenesis which is not completely clear. It will be significant to find new pathogenesis or target for RA research.

Circular RNA (circRNA) is a unique type of endogenous RNA. It does not have free 3 ′or 5′ ends, but forms covalently closed continuous rings. It is characterized by conserved interspecies sequence, stable structure, and specific expression of cells or tissues (Luo et al., 2020). In the past decades, it has been confirmed that circRNA is involved in the pathogenesis of many diseases, including diabetes (Kristensen et al., 2019), neurodegenerative disease (Verduci et al., 2021), cancer (Liu K-S et al., 2019), and cardiovascular disease (Altesha et al., 2019). CircRNAs are highly expressed in human islets, and some are regulated differently in the islets of type 2 diabetic donors (Haque et al., 2020). Differential expression of circRNAs in brain tissue is associated with Alzheimer’s disease. It is a biomarker and regulator, and also a potential therapeutic target (Lo et al., 2020). Several studies have confirmed that many circRNAs are involved in the pathogenesis of RA. For example, the altering effect of circRNA_09505 on inflammation is investigated *in vitro* and *in vivo* by use of macrophage cell models and collagen-induced arthritis (CIA) mice, elevated circRNA_09505 promotes arthritis and inflammation (Ouyang et al., 2021). A growing amount of research shows that circRNA has a close relationship with RA.

In the field of biomedicine, more and more circRNA have been found to play an important role in various diseases, and its expression changes, regulatory functions, and action mechanisms in the pathogenesis and development have attracted more and more attention (Patop and Kadener, 2018; Liu Z et al., 2019). In this review, the functional role of circRNAs in the progress of RA in recent years is summarized, which is expected to provide a reference for revealing the pathogenesis of RA. This paper mainly discusses the newly discovered circRNAs in inflammatory immune response, hoping to provide help for future research in RA.

## 2 Overview of rheumatoid arthritis

RA is a systemic inflammatory disorder that mainly affects the diarthrodial joint. It is the most common form of inflammatory arthritis and has a substantial societal effect in terms of cost, disability, and lost productivity. RA varies greatly in age, sex, race, and region. The global incidence rate of RA is 0.5%–1% (Safiri et al., 2019). But so far, RA still can not be cured, which may be related to unclear pathogenesis.

Many previous studies have suggested that the pathogenesis of RA is complex, and its occurrence and development may be closely related to heredity, the surrounding environment, the disorder of immune function, and platelet activation. The popular view is that RA results from interacting with antigen-presenting cells (APC) and CD4^+^ cells. APC presents complex primary histocompatibility complex class II molecules (MHC-II) and antigen polypeptides, which bind to T cell surface receptor (TCR). Then macrophages are activated and secrete proinflammatory cytokines such as IL-1 and IL-α. These cytokines activate synovial fibroblasts and chondrocytes around articular cartilage to secrete various enzymes that degrade glycoproteins and collagen, resulting in tissue destruction (Takeshita et al., 2019). Therefore, it is of great significance for the treatment and prognosis of the disease to explore the etiology of RA and find the biomarkers for diagnosing the disease. Inflamed synovium is central to the pathophysiology of RA. Several lines of evidence implicated the participation of T cells in the pathogenesis of RA. Secretions of Macrophages, especially TNF-α and IL-1β, are considered critical inflammatory mediators. These immune cells stimulate synovium, cause inflammation, and cause synovium surface roughness and erosion. Moreover, osteoclasts are overactivated, leading to bone loss and joint destruction in RA patients.

The main purpose of the treatment of rheumatoid arthritis is to avoid joint destruction through early and effective anti-inflammatory treatment. But so far, RA can not be cured, which may be related to its pathogenesis. Therefore, it is of great significance for treatment and prognosis to explore the etiology and find the biomarkers for diagnosis.

## 3 Overview of circular RNAs

Non-coding RNA (ncRNA) is a unique RNA transcript, accounting for more than 90% of RNA in the human genome. Except for a few with open reading frames and thus coding potential, ncRNA usually does not encode proteins but acts as an essential regulator of various biological processes such as developmental proliferative transcription, post-transcriptional modification, apoptosis cell metabolism, etc (Slackj and Chinnaiyan, 2019; Wang et al., 201927). CircRNA is a kind of special closed-loop endogenous ncRNA. It is formed by RNA polymerase transcription after precursor mRNA variable shearing processing, because circRNA has strong intracellular stability, high conservatism, and tissue specificity sex, with transcriptional and post-transcriptional regulation and the ability to be translated into proteins, and then participates in the occurrence and development of a variety of diseases (Yin et al., 2019).

At present, divide according to the source and constituent sequence of the genome, the sources, and sequences of RNA are divided into exon circRNA (ECIRC RNA), intron circRNA (ciRNA), and circRNA (Elci RNA) jointly formed by them, ecIRC RNA, the last category is circRNA from within and between genes (Jeck et al., 2013). It mainly exists in the cytoplasm, driving the formation of circRNA composed of two exons or intron base complementary pairing to form a loop, and then shearing intron to form cirRNA. In this process, ciRNA can be formed by intron lasso escaping from branches ElciRNA formation may be driven by intron base complementary pairing to form ecircRNA while preserving intron sequences.

**FIGURE 1:**
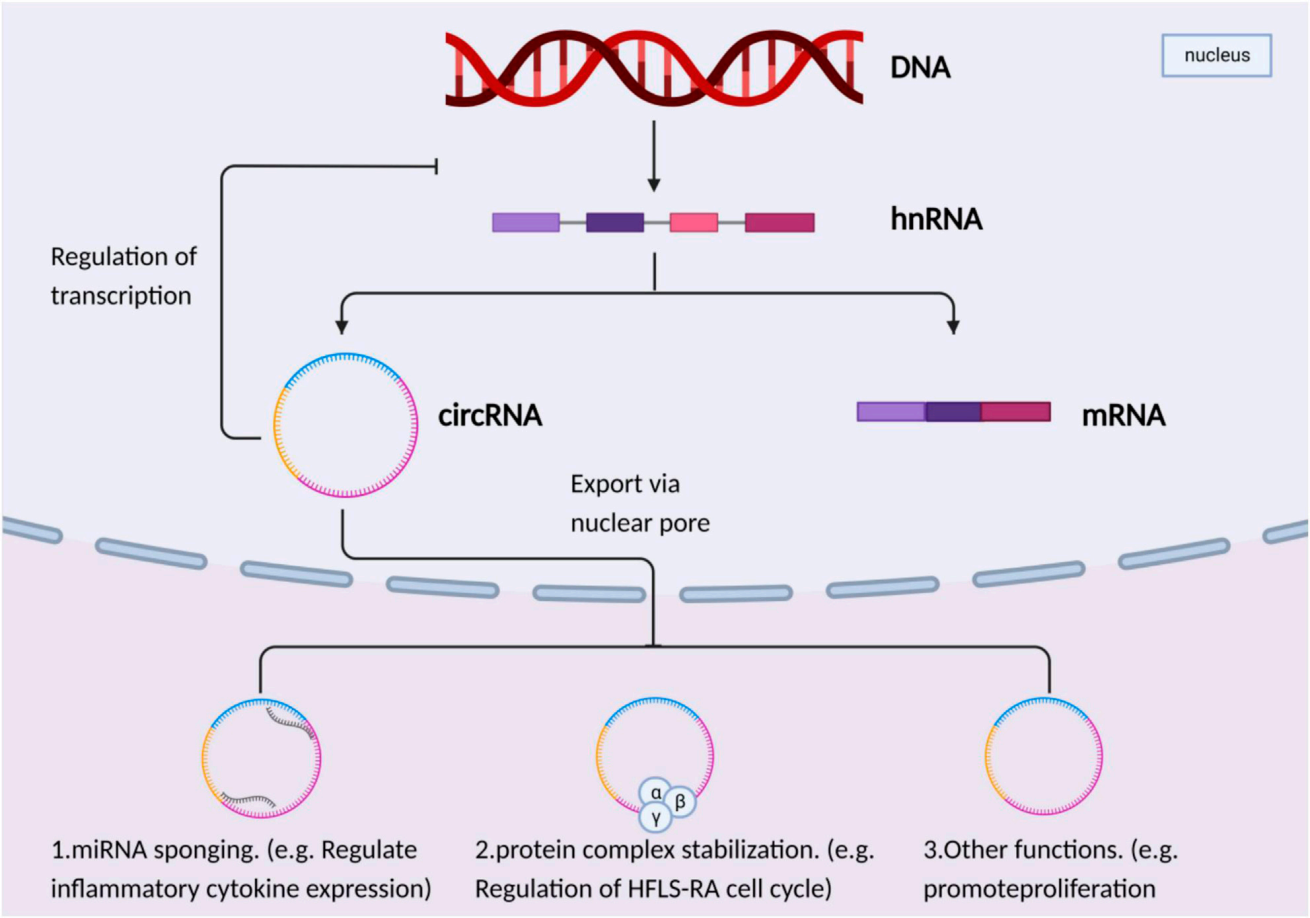
CircRNAs act as miRNA sponges and affect the occurrence and development of RA by regulating the activation of NF-κB signaling pathway, JAK-STAT signaling pathway and MAPK signaling pathway.

Biological functions of circRNA (Figure 1): 1)miRNA sponge function circRNA acts as endogenous RNA to competitively bind and inhibit miRNA, thus regulating the expression of downstream target genes (Tay et al., 2014). 2) Interaction with RNA binding protein (RBP). CircRNA binds to RBP to form a complex, and plays a role in the stability and localization of circRNA splicing, replication, folding, and thus affecting the occurrence of cell cycle apoptosis (Jeck and Sharpless, 2014). 3) Translation into proteins or peptides: usually the case with circRNA It cannot be translated into proteins, but can be translated into proteins when circRNA contains internal ribosomal binding sites (Pamudurti et al., 2017). 4) To regulate gene transcription, circRNA is abundant in the eukaryotic transcriptome. EIciRNA can form a complex through interaction with RNA, which further interacts with RNA polymerase and finally regulates the expression level of parental genes in a cis-mode. 5) Regulating the variable and shearing of RNA, the expression level of circRNA can regulate the shearing function of its mRNA.

In recent years, due to the development of high-throughput sequencing technology and various bioinformatics technologies, more and more circRNAs have been found to play an important role in various diseases. It is involved in the pathogenesis of many human diseases, including tumors, gynecological diseases, and autoimmune diseases. CircRNA may become a biomarker for diagnosing various difficult diseases or a target for curing diseases. For example, circRACGAP1 regulated the AKT signaling pathway *via* binding to miR-22 p–3 p in the progression of Systemic lupus erythematosus (SLE), suggesting therapeutic targets for SLE treatment (Mei et al., 2022). CircRNA may have a similar mechanism to RA. The following is a summary of the research progress on the relationship between circRNA and RA.

## 4 Function role of circular RNA in rheumatoid arthritis

Numerous studies have shown that circRNAs are differentially expressed in the occurrence and development of RA. The expressions level of circRNA_092516, circRNA_104871,circRNA_003524, circRNA_101873,circRNA_103047,circRNA_0008410 and circRNA_0088036 (Ouyang et al., 2017; Luo et al., 2020; Yang et al., 2022a) in peripheral blood mononuclear cells (PBMC) of RA patients were significantly increased, especially circRNA_0140271 is highly expressed in female RA (Chen et al., 2021a), while circRNA_0000396, circRNA_0130438, circRNA_0000175 and circRNA_101328 (Lu et al., 2022) were decreased in PBMC of RA patients. Circ_0002715, circ_0001947, circ_0000367, and circ_0035197 in peripheral blood of patients with new onset RA increased significantly (Luo et al., 2019; Yang et al., 2022b), and circ_0044235 (Chen et al., 2021b) decreased significantly. Circ_0003972, circ-AFF2, circ_0003353, and circ_0088194 (Feng et al., 2022; Wen et al., 2022) are highly expressed in RA patients and fibroblast-like synoviocytes of rheumatoid arthritis (HFLS-RA) cells, circ_0008360, and circ_0130438 expressions were down-regulated. Circ_0005008, circ_0005198, and circ_0000175 were confirmed to be elevated in plasma samples from patients with new-onset RA, while circ_0044235 was significantly reduced.

Circ_0003972 is located at chromosome 9. It was mainly present in the cytoplasm of HFLS-RA cells. Circ_0003972 was significantly upregulated in HFLS-RA cells (Li et al., 2022a). It was mainly present in the cytoplasm of HFLS-RA cells. Circ_0003972 knockdown inhibited proliferation and inflammation in TNF-α-induced HFLS-RA cells. Moreover, TNF-α is an important multifunctional inflammatory cytokine involved in regulating cell apoptosis, survival, and immune response (Hu et al., 2022). Changes in the concentration of TNF-α in the inflammatory microenvironment can affect cell proliferation, differentiation, apoptosis, and other cell functions. TNF-α may affect the changes of intracellular factors in synovial fibroblasts by binding to the corresponding cytokine receptors on the cell surface. Circ_0003972 knockdown abolished the promotion effect of TNF-α on the cell cycle in HFLS-RA cells.

Circ_0000396 is located at chromosome12. It was decreased in RA synovial tissues and synovial fibroblasts of RA (RASF) (Wang et al., 2021). RASF stimulation is a pivotal factor in the transformation of affected synovium to healthy synovium, thus inducing the expansion of arthritis and distant joint destruction. RASF proliferation also results in immunoreaction and ultimately contributes to joint damage. The secretion of IL-6, IL-1β, IL-8, and TNF-α inflammatory cytokines was suppressed by overexpressed circ_0000396, thus suppressing inflammation in RASF.

**TABLE 1:** The aberrantly expressed circRNAs in RA.

CircRNA	Express	Tissue	Model	Species	References
circRNA_092516	↑	PBMCs	Blood samples	Human	Ouyang et al. (2017)
circRNA_104,871	↑	PBMCs	Blood samples	Human	Ouyang et al. (2017)
circRNA_003524	↑	PBMCs	Blood samples	Human	Ouyang et al. (2017)
circRNA_101,873	↑	PBMCs	Blood samples	Human	Ouyang et al. (2017)
circRNA_103,047	↑	PBMCs	Blood samples	Human	Ouyang et al. (2017)
circRNA_0008410	↑	PBMCs	Blood samples	Human	Luo et al. (2020)
circRNA_0088036	↑	PBMCs	Synovial cell model	Human	Yang et al. (2022a)
circRNA_09505	↑	PBMCs	Macrophage cell models/Mouse model	Human/Mouse	Yang et al. (2020)
circRNA_0000396	↓	PBMCs	whole blood	Human	Wang et al. (2021)
circRNA_0130,438	↓	PBMCs	whole blood	Human	Wang et al. (2021)
circRNA_0000175	↓	PBMCs	Blood samples	Human	Lu et al. (2022)
circRNA_101,328	↓	PBMCs	Blood samples	Human	Lu et al. (2022)
circRNA_0002715	↑	peripheral blood	Blood cell model	Human	Luo et al. (2019)
circRNA_0001947	↑	peripheral blood	Synovial cell model	Human	Yang et al. (2022b)
circRNA_0035197	↑	peripheral blood	Blood cell model	Human	Luo et al. (2019)
circRNA_0044235	↓	peripheral blood/plasma	Blood cell model	Human	Chen et al. (2021b)
circRNA_0003972	↑	HFLS-RA	HFLS-RA cell model	Human	Wen et al. (2022)
circRNA_0003353	↑	HFLS-RA	Cell model	Human	Wen et al. (2022)
circRNA_0088194	↑	HFLS-RA	Cell model	Human	Feng et al. (2022)
circRNA_0130,438	↓	HFLS-RA	Cell model	Human	Lu et al. (2022)
circRNA_0005008	↑	plasma	Cell model	Human	Ouyang et al. (2021)
circRNA_0005198	↑	plasma	Cell model	Human	Ouyang et al. (2021)
circRNA_0000175	↑	plasma	Cell model	Human	Lu et al. (2022)

Circ0088036 is a 279-bp transcript originating from the Sushi Domain Containing 1(SUSD1) gene (Zhong et al., 2020). There is definite research confirmation that circ_0088036 is significantly elevated in PBMC in patients with RA. Recently, Some studies have discussed the role of circ0088036 in the pathogenesis of RA. The findings show that circ_0088036 expression is significantly increased in RA-FLSs. Circ_0088036 regulates the proliferation and migration of RA synovial fibroblasts by affecting the expression of SIRTl, while SIRTl can regulate the inflammatory development, angiogenesis, articular cartilage, and bone erosion of RA. Functionally, the upregulation of circ_0088036 promotes the proliferation and migration of RA-FLSs. In terms of mechanism, circ_0088036 acts as a miR-140 p–3 p sponge to up-regulate SIRT 1 expression, thereby promoting the progress of RA.

Circ_0130438 is located at chromosome6 and from the gene Karyopherin Subunit Alpha 5 (Li et al., 2022b). The latest discovery shows the significant reduction of circ_0130438 in RA tissues and RA-FLSs. TNF-α downregulates circ_0130438 expression in human fibroblast-like MH7A synoviocytes. Moreover, circ_0130438 represses TNF-α-induced cell function change *via* its ceRNA activity. Additionally, apart from TNF-α, data also showed the suppression of other inflammrelated factorsactors (IL-1β, IL-17A, and LPS) on circ_0130438 expression in MH7A cells, suggesting the implication of circ_0130,438 in human inflammatory diseases.

Circ_09505 was mainly localized in the cytoplasm of macrophages (Yang et al., 2020). It is one of the most aberrantly expressed CircRNAs in RA. The expression of circRNA_09505 was positively associated with ESR, CRP, and RF levels in serum from RA patients. CircRNA_09505 contributes to RA by regulating macrophage proliferation, cell cycle, and inflammatory response. CircRNA_09505 could function as a miR-6089 sponge in macrophages. Yang et al. investigated the influence of circRNA_09505/miR-6089 on macrophage-mediated inflammation in RA pathogenesis. CircRNA_09505 aggravates macrophage inflammation by promoting the generation of inflammatory cytokines TNF-α, IL-6, IL-8, IL-12, and IL-1β, which are the most commonly dysregulated cytokines in RA. Inflammation and immunological disorders caused by macrophage, B, and T lymphocytes contribute to RA.

The expression levels of circ_0005008 and circ_0005198 in patients with new onset RA were significantly higher than those in patients with HC and new onset SLE (Ouyang et al., 2021). This suggests that plasma circ_0005008 and circ_0005198 may play a role in the pathogenesis of RA. In addition, the expression levels of circ_0005008 and circ_0005198 in plasma of patients with new onset RA are related to DAS28, ESR, CRP, and RF, reflecting the severity of disease activity. Therefore, plasma circ_0005008 and circ_0005198 levels may be a biomarker of systemic inflammation and disease severity in RA. However, their expression level has nothing to do with anti-CCP, which reflects the prognosis of RA patients, indicating that plasma circ_0005008 and circ_0005198 are not potential biomarkers of patient prognosis.

In general, CircRNA is a new field in the study of the pathogenesis of RA. It is a kind of functional biological macromolecules that can participate in the pathogenesis of RA through a variety of general pathways, including promoting the continuous proliferation of synovial cells, chronic inflammation, fibroid changes, cytokine imbalance, and the formation of pannus, the destruction of cartilage and subchondral bone. It can also be used as a biomarker of peripheral blood of RA. CircRNA is a kind of functional biological macromolecule. CircRNA has its specific expression pattern in tissues and cells. Therefore, exploring the expression of different CircRNA in RA may provide new markers and therapeutic targets for RA.

## 5 Special emp in of rheumatoid arthritis

Cytokines (CK) are mostly small molecule polypeptides, proteins, or glycoproteins. In autoimmune diseases such as RA, they are mainly produced by activated immune cells with high activity and different functions. Inflammatory cytokines are the main mediators of RA inflammatory response, when the effect of pro-inflammatory factors in inflammatory cytokines is higher than that of anti-inflammatory factors, it will lead to multi-system immune complications.

A large number of studies have confirmed that cytokines are directly related to RA (Abdollahzad et al., 2015), such as TNF-α, IL-1β, IL-6, IL-8, IL-12, etc. In RA, TNF-α activates synovial fibroblasts, promotes epidermal proliferation, and recruits inflammatory cells. In RA, TNF-α Activates synovial fibroblasts, promotes epidermal proliferation, and recruits inflammatory cells. Various cytokines including IL-1β, IL-6, and TNF-α upon activation, synovial fibroblasts overexpress cathepsin and matrix metalloproteinase (MMP), followed by collagen and proteoglycan breakdown, cartilage and bone destruction, and finally joint erosion (Li et al., 2022c). It is reported the expression of circRNA_09505 is upregulated in PBMCs from patients with RA and promotes the production of TNF-α, IL-6, and IL-12 through the ceRNA mechanism. The proliferation and cell cycle are significantly promoted when circRNA_09505 is upregulated in macrophages. New research shows that The level of circRNA circ_0130438 was reduced in RA tissues and FLSs isolated from RA tissues. Circ_0130438 enhanced KLF9 expression in TNF-α-stimulated MH7A cells by functioning as a competing endogenous RNA (ceRNA) for miR-130a-3p. The elevated expression of circRNA circ_0130438 suppressed the TNF-α-induced proliferation and migration of MH7A cells, as well as their pro-inflammatory cytokines (IL-1β, IL-6, and IL-8) production.

A variety of immune cells participate in and mediate autoimmune inflammation in RA, including T, B lymphocytes, macrophages, neutrophils, etc. Macrophages are essential cells that promote systemic autoimmune disorders and chronic joint inflammation by linking innate and adaptive immunity in RA (Wang et al., 2022a). An increased level of macrophages in inflammatory lesions is a critical feature of RA, which infiltrates synovial tissues and causes joint erosion. CircRNA_ 09505 is the most significantly increased circRNA in RA PBMCs, which can function as a miR-6089 sponge and regulate macrophage inflammation by targeting AKT1 *in vitro*. In addition, circRNA_09505 in macrophages were blocked can reduce inflammation and joint injury in collagen-induced arthritis (CIA) mice *in vivo*.

Many differentially expressed circRNAs genes should be further verified *in vivo* and *in vitro* to affect the inflammatory response of RA in different ways, providing theoretical support for the development of new RA biomarkers and molecular targets.

## 6 Regulate mechinching of circular RNAs in rheumatoid arthritis

**FIGURE 2:**
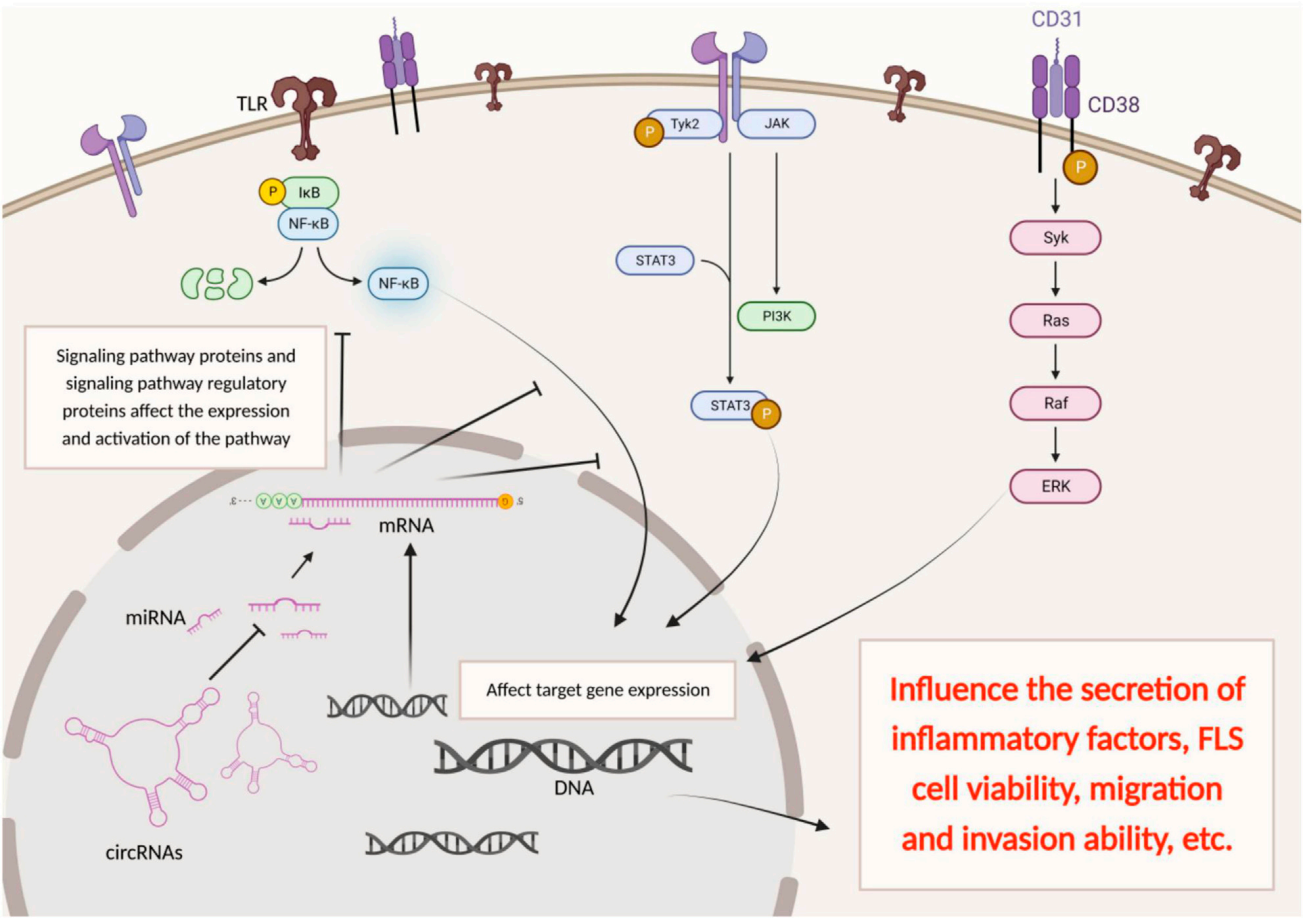
CircRNAs have multiple functions and can be involved in the pathogenesis of RA through multiple pathways.

As we all know, the cell signaling pathway is the most microscopic and deepest biological regulatory mechanism. Knowledge of a disease-related signaling pathway facilitates the search for reliable biomarkers and valuable drug candidate targets. This part mainly summarizes the cellular signaling pathways involved in the regulation of circRNAs in the process of rheumatoid arthritis (Figure 2).

NF-κB is a protein complex that controls transcribed DNA, cytokine production, and cell survival. The NF-κB family has five members, including nf-kb1 (P50), nf-kb2 (p52), rela (p65), RelB, and c-Rel. An NF-κB signaling pathway is ubiquitous in eukaryotic cells because it can interact with the enhancer of B cell immunoglobulin K light linked gene κB sequence-specific binding. Studies have shown that the NF-κB signaling pathway plays an important role in the development of articular cartilage and osteoarthritis. It participates in the body’s inflammatory response, and immune response, and even can regulate apoptosis and stress response. Excessive activation of NF-κB is closely related to many inflammatory diseases in humans (Raskatov et al., 2012; Zusso et al., 2019). Yang et al. found that circRNA_09505 could function as a miR-6089 sponge to regulate inflammation in collagen-induced arthritis (CIA) mice through the AKT1/NF-κB axis. Specifically, circRNA_09505 aggravated macrophage inflammation by promoting the production of inflammatory cytokines TNF-α, IL-6, IL-8, IL-12, and IL-1β through the miR-6089/AKT1/NF-κB axis, which is the most commonly deregulated cytokines in RA.

The JAK-STAT signaling pathway is mainly composed of three parts: cell surface receptors, the JAK protein family, and the STAT protein family. The JAK family can selectively bind to the membrane-proximal domain to form different combinations of receptors. These receptors form homologous or heterodimers after binding with ligands to initiate signal transduction and directly regulate the transcription of target genes. JAK2-STAT3 is the main member of the JAK-STAT signaling pathway family. It is important for initiating innate immunity, coordinating adaptive immune mechanisms, and ultimately suppressing inflammation and immune responses (Banerjee et al., 2017; Xin et al., 2020). Rheumatoid arthritis fibroblast-like synoviocytes (RA-FLS) are another important cell besides macrophages that play an important role in the pathological progression of RA. It can not only secrete inflammatory factors like macrophages but its migration and invasion can also cause the destruction of bone tissue and aggravate the pathological damage of RA. However, triptolide (TPL) can regulate circRNA_0003353, interfere with JAK2/STAT3 signaling pathway, inhibit the inflammatory response and cell migration of RA-FLS, and play a role in the treatment of RA (Wang et al., 2022b).

MAPK access has four main branch routes: ERK, JNK, p38/mapk, and ERK5. JNK and p38 have similar functions, which are related to inflammation, apoptosis, and growth; ERK is mainly responsible for tube cell growth and differentiation. ATF2, a defined downstream target of ERK cascade, belongs to the activator protein-1 (AP-1) transcription factor family. Immunohistochemistry assay confirmed ATF2 overexpression in synovial sections of RA patients. ATF2 knock-down led to a decrease of IL-6, IL-1β, and MMP-13 expression and inhibited the cell migration and invasion in SW982 cells after TNF-α induction. Researchers compared the mRNA expression of rheumatoid arthritis patients and healthy volunteers through the GEO database and used a functional enrichment algorithm to map the interaction group of circRNAs-miRNAs-mRNAs. Loss-of-function and rescue analyses of candidate circRNAs were performed *in vitro*. They found CirRNA Hsa_circ_0001859 acted as a miRNA sponge to compete with ATF2 for miR-204/211 and thus elevated ATF2 expression and promoted inflammatory activity in SW982 cells (Li et al., 2018).

In conclusion, circRNAs often act as miRNA sponges and participate in the regulation of various cellular signaling pathways, regulating the pathological progression of RA by affecting the secretion of inflammatory factors, FLS cell viability, migration and invasion ability, etc. These cellular signaling pathways are conducive to our in-depth understanding of the mechanism of circRNA regulating RA, and also provide us with more accurate ideas and directions for the development of relevant target drugs.

## 7 Poteional chilnic apply of circularRNAs in rheumatoid arthritis

Rheumatoid Factor (RF), anti-cyclic Citrullinated peptide antibody, Erythrocyte sedimentation rate (ESR), and erythrocyte sedimentation rate (ACCPA) are considered traditional molecular markers for the diagnosis of RA (Tavasolian et al., 2018). However, they lack specificity and are of low priority. With the improvement of biological techniques, more specific molecular markers reflecting RA have been discovered. Multiple studies have shown that circRNA is differentially expressed in the occurrence and development of RA. Therefore, the identification of new and promising rheumatoid arthritis biomarkers is crucial for the early diagnosis and treatment of rheumatoid arthritis.

CircRNAs are widely expressed especially in the synovial tissue of patients with rheumatoid arthritis. And circRNA has been measured in tissue, serum, exosomes, and other body fluids in various diseases. Microarray analysis revealed a specific circRNA expression profile in RA, and that revealed that the expression levels of circRNA_104,194, circRNA_104593, circRNA_103334, circRNA_101407, and circRNA_102594 (Zheng et al., 2017) were significantly abnormal by means of RT-qPCR in RA patients. Meantime, Then 35 RA and 30 normal subjects were collected for real-time quantitative PCR verification, ROC curve analysis showed that circRNA_104871 had a significant diagnostic value of RA. Circ_003524, circRNA_101873, and circRNA_103047 indicate that circRNA can be used as a potential biomarker for the diagnosis of RA patients. It has been shown that HAS-CIRC-0001859 is recognized in synovial tissue, this suggests that it could be used as another diagnostic marker for RA. Circ_0044235 (Chen et al., 2021b) locates at chromosome 17 and is spliced from the cell division cycle 27 gene. It was found that compared with HC, the expression level of circ_0044235 in peripheral blood from RA patients was down-regulated significantly (*p* < 0.0001). And the AUC of circ_0044235 in peripheral blood was as high as 0.779. But it was not correlated with biomarkers for disease severity. Receiver operating characteristic (ROC) curve analysis suggested that the circ_0044235 in peripheral blood has a significant value in diagnosing RA. The study indicates that the circ_0044235 in peripheral blood may be a potential biomarker of patients with RA.

In addition, plasma circ_0000175 and circ_0044235 expression levels were associated with disease activity and severity of RA. There are also research findings that HSA-CIRC-0044235 in peripheral blood can specifically identify RA patients with higher diagnostic accuracy Also, analysis using ROC curves implied that circ_0005008 and circ_0005198 have significant value in the diagnosis of RA. The plasma expression levels of circ_0005008 and circ_0005198 in new-onset patients with RA both correlated with DAS28, ESR, CRP, and RF, which reflect the severity of disease activity. Thus, plasma levels of circ_0005008 and circ_0005198 might serve as biomarkers for RA systemic inflammation and disease severity. Moreover, the plasma levels of circ_0005008 and circ_0005198 had AUCs of 0.829 and 0.783 to show high specificity and sensitivity, which indicated that they have good potential as RA diagnostic biomarkers.

Some scholars (Avenoso et al., 2021) studied the level of miR-146a and its correlation with inflammatory mediators in the experimental model of inflammatory response induced by 6-mer HA in human cultured chondrocytes. The results showed that chondrocytes receiving miR-146a mimics and 6-mer HA significantly reduced inflammatory cytokines and MMP-13, while chondrocytes exposed to miR-146a inhibitors and 6-mer ha increased destructive cytokines and MMP13. The expression of CD44 receptor is not affected by miR-146a treatment, In contrast, TLR-4 expression and NF-kB activation are changed, which may help to develop new treatment strategies to reduce the incidence rate of OA and RA.

Angiogenesis and the interaction between fibroblast-like synoviocytes (FLS) and human dermal microvascular endothelial cells (HDMECs) *via* vascular endothelial growth factor (VEGF) angiogenic functional modules play a critical role in RA disease progression. It has been reported that circ_0000284 (Zhang et al., 2021) could function as a miRNA sponge to regulate vascular dysfunction and vessel growth. Fluorescence *in situ* hybridization (FISH) analysis showed that circ HIPK3 (Saad El-Din et al., 2022) was located mostly in the cytoplasm of RA-FLS. Zhang et al. (2021) Found that circHIPK3 should play a central role in abnormal angiogenesis in the inflammatory microenvironment, and tried to use it as a key target for the treatment of RA. Zhang et al. (2021) established the collagen-induced arthritis (CIA) mouse model. After the injection of arsenic trioxide (ATO), the inflammation, cartilage, and bone destruction of mice were significantly reduced in the knee joint slices of CIA mice. This prompt ATO may be a potential therapeutic strategy for RA.

CircRNAs not only represent possible targets of RA but may also be used as effectors for targeting miRNA/siRNA. Circular RNAs are much more stable than mRNAs and may produce proteins. Therefore, exogenous circRNAs themselves can serve as vectors for gene delivery, providing robust and stable protein expression in eukaryotic cells (Li et al., 2022b). Therefore this circRNA represents a biomarker with potential applications in RA diagnosis and therapy. With the development of gene therapy technology, the currently rapidly developing RA treatment has the potential to open up a new dimension. Nevertheless, some gene therapy trials have had some success, but experimental and genetic research has yielded new targe.

## 8 Future perspectives

Gene editing technology refers to a new technology that uses nucleases to modify target genes at specific sites to achieve specific DNA knock-out, knock-in, and mutation, and ultimately down-regulate or up-regulate gene expression so that cells can obtain new phenotypes. Recent studies have found that RNA base editing has the potential to replace genome editing and become one of the ways of gene therapy. CRISPR/cas9-sgRNA MMP-13 can inhibit the catabolism of the cartilage matrix, but it cannot target and recognize chondrocytes. Using genetic engineering technology, through the transformation of exosome membrane protein lamp-2b, the modified hybrid exosomes can target the delivery of sgRNA MMP-13 into articular chondrocytes, inhibit the catabolism of articular cartilage matrix, and achieve effective treatment of OA. At present, some scholars overexpress TGF by using gene editing in the mouse model of damaged cartilage-β amniotic mesenchymal stem cells, exploring the protection of cartilage and the treatment of inflammatory arthritis. In the treatment of RA, Jing et al. (2015) used a miR-155 knockout RAW 264.7 macrophage cell line and found that in the cell line, SHP1 was up-regulated and the pro-inflammatory cytokine-making process was impaired. This proves that genome editing may be a potential treatment strategy for RA. Although the molecular action and regulatory mechanism of many circRNA related to rheumatoid arthritis have not yet been revealed, aiming at the structural characteristics and action mechanism characteristics of circRNA, using gene editing technology could lead to this functional circRNA and related genes playing a critical role in guiding pathophysiology.

The use of nano drugs can achieve high drug concentrations in target tissues while avoiding unrelated tissues. This treatment will be much better than the equivalent dose given as free pro-drugs. It can use a lower total amount of drugs and administration frequency, thereby reducing side effects. The institutions have verified through experiments that compared with free precursor drugs, nano drugs show superior pharmacokinetics, biodistribution, tolerability, and therapeutic efficacies in the treatment of inflammatory diseases. According to the special pathological characteristics of RA, more intelligent drug delivery systems with multiple functions can be deeply studied, such as selective accumulation, intelligent drug release, and active targeting of certain cell types. For example, nano drugs are engineered with small molecules, polymers, peptides, or proteins to achieve active targeting. Therefore, we can expect to realize the intelligent design and precise regulation of circRNA nano drugs through an in-depth study of the role of circRNA in the inflammatory and immune process of RA and the prospects in the field of biomedical materials science and to realize the synergistic treatment of RA by combined administration with other drugs.

## 9 Conclusion

RA is an autoimmune disease that needs early diagnosis and treatment. Accumulating evidence has established that circRNA plays an essential role in the pathogenesis of RA. In this review, we discuss the newly discovered circRNA signaling pathways. We learn that circRNA often acts as miRNA sponges and participates in the regulation of various cellular signaling pathways, regulating the pathological progression of RA by affecting the secretion of inflammatory factors, FLS cell viability, migration and invasion ability, etc. Although a series of published studies have revealed the role of circRNA in the regulation of inflammation and autoimmune by multiple signaling pathways in RA, the research on its regulatory mechanism is still unclear. With the development of next-generation sequencing and other modern molecular biology technologies, it is reasonable to believe that more circRNA molecules regulating the inflammatory immune mechanism and related targeted drugs will be revealed.
